# *CCDC88C::PDGFRB*-rearranged myeloid neoplasm with predominant neutrophilia and rapid response to imatinib: a molecularly defined case report

**DOI:** 10.3389/fonc.2026.1855322

**Published:** 2026-06-22

**Authors:** Yafei Guo, Huiqiang Wu, Zhiyin Cai, Genwang Chen, Meiling Li, Xizhe Guo

**Affiliations:** 1 Department of Hematology, the Second Affiliated Hospital of Fujian Medical University, Quanzhou, Fujian, China; 2 Department of Clinical Laboratory, the Second Affiliated Hospital of Fujian Medical University, Quanzhou, Fujian, China

**Keywords:** CCDC88C, gene fusion, imatinib, myeloproliferative neoplasm, PDGFRB, RNA sequencing

## Abstract

Myeloid/lymphoid neoplasms with *PDGFRB* rearrangements are rare hematologic malignancies usually associated with eosinophilia and sensitivity to tyrosine kinase inhibitors, whereas atypical presentations remain poorly defined. We report a 60-year-old man with persistent leukocytosis and thrombocytopenia detected during a routine health examination. Bone marrow examination revealed marked granulocytic proliferation without increased blasts, and peripheral blood showed predominant neutrophilia with only mild eosinophilia. Cytogenetic analysis identified a t(5;14) translocation. RNA sequencing detected an in-frame *CCDC88C exon 12::PDGFRB* exon 11 fusion, and targeted sequencing additionally identified an *ATM* frameshift mutation. Treatment with low-dose imatinib (200 mg daily) led to rapid normalization of leukocyte counts within one week and sustained hematologic remission during follow-up. This case broadens the clinicopathologic spectrum of *CCDC88C::PDGFRB*-rearranged neoplasms and highlights the value of RNA sequencing in identifying actionable kinase fusions in atypical myeloproliferative presentations.

## Introduction

Myeloid/lymphoid neoplasms with eosinophilia and rearrangements of platelet-derived growth factor receptor beta (*PDGFRB*) represent a distinct group of hematologic malignancies characterized by constitutive activation of tyrosine kinase signaling. These neoplasms are generally linked to eosinophilia and exhibit a high level of sensitivity to tyrosine kinase inhibitors such as imatinib. However, mounting evidence indicates considerable heterogeneity in their clinical presentation (1, 2).

To date, numerous *PDGFRB* fusion partners have been identified, contributing to aberrant activation of downstream signaling pathways (3, 4). However, the majority of reported cases remain limited to isolated case reports, and rare fusion partners continue to be identified (5–7). The clinical manifestations of this condition can vary widely, and atypical presentations without prominent eosinophilia may lead to diagnostic challenges. Among these rare fusion partners, *CCDC88C* has only rarely been reported in *PDGFRB*-rearranged myeloid neoplasms, and the associated clinicopathologic spectrum remains poorly defined.

In this study, we present a rare case of a *PDGFRB*-rearranged myeloid neoplasm that harbours a *CCDC88C::PDGFRB* fusion, as detected by RNA sequencing. The patient had predominant neutrophilia, mild eosinophilia, and thrombocytopenia, but remained clinically asymptomatic. In addition, a concurrent *ATM* frameshift mutation was detected, raising the possibility of additional molecular complexity. The patient showed a rapid hematologic response to imatinib therapy, underscoring the significance of molecular diagnosis in directing treatment.

## Case presentation

A 60-year-old male patient was admitted to our hospital after abnormal blood cell counts were detected during a routine health examination. The patient exhibited no symptoms, including fever, fatigue, or weight loss. Preliminary laboratory findings on December 18, 2025 revealed a total leukocyte count of 24.92×10^9/L, a neutrophil count of 21.41×10^9/L, an eosinophil count of 0.72×10^9/L, a basophil count of 1.24×10^9/L (5% on manual differential), a hemoglobin level of 164 g/L, and a platelet count of 77*10^9/L. Peripheral blood smear analysis revealed a predominance of mature neutrophils with a left shift (manual differential: band forms 23%, segmented neutrophils 56%, metamyelocytes 2%, promyelocytes 1%, lymphocytes 6%, monocytes 1%, eosinophils 6%, basophils 5%). Serum lactate dehydrogenase was mildly elevated at 240 U/L (reference <250 U/L). On January 21, 2026, the WBC count peaked at 32.21×10^9/L, accompanied by mild to moderate eosinophilia (up to 1.93×10^9/L) and basophilia (1.93×10^9/L).

Physical examination revealed no palpable splenomegaly. However, abdominal ultrasonography showed mild splenic enlargement, with a thickness of 4.1 cm.

**Figure 1:**
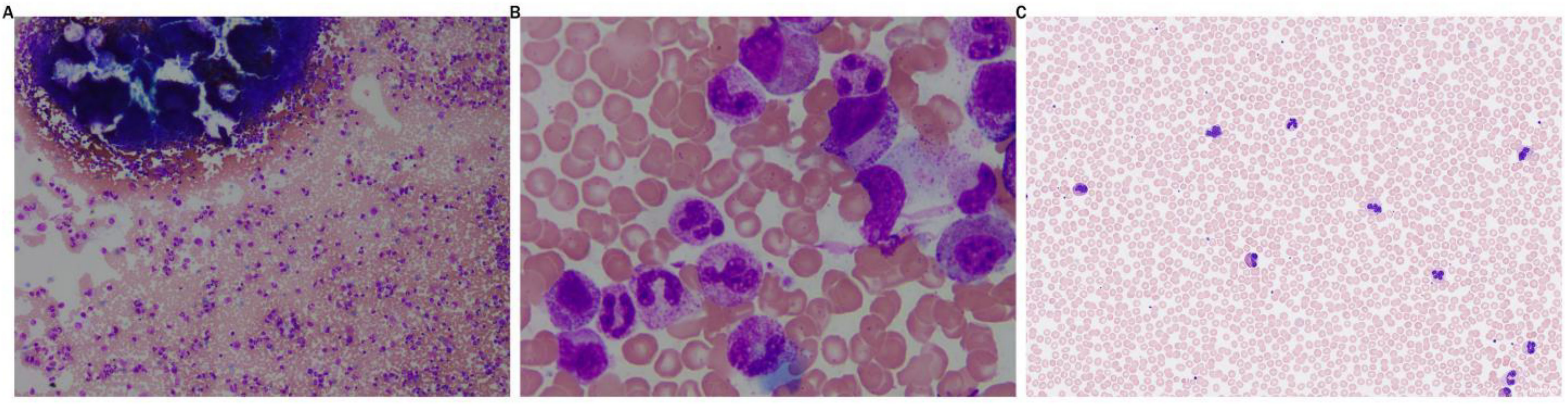
Morphology of bone marrow and peripheral blood. **(A)** Bone marrow smear, low-power magnification (×100) showing markedly hypercellular bone marrow with extensive granulocytic proliferation. **(B)** Bone marrow smear, high-power magnification (×1000, oil immersion) demonstrating predominant neutrophilic granulopoiesis with increased myelocytes and metamyelocytes; no increase in blast cells is observed. **(C)** Peripheral blood smear, high-power magnification (×1000, oil immersion) showing marked neutrophilia with left shift; eosinophils are only occasionally seen.

Bone marrow aspirate smears were of good quality and showed extremely active proliferation. Granulopoiesis was markedly increased (88.5%), whereas erythropoiesis was relatively decreased (6.5%), with a G/E ratio of 13.62:1. The proportion of neutrophilic myelocytes and metamyelocytes was increased, with coarse and increased cytoplasmic granules. Erythroid cells were reduced in proportion, without obvious morphologic abnormalities in mature erythrocytes. Lymphocytes were decreased in proportion, and monocytes were within the normal range. A total of 102 megakaryocytes were observed, but platelet-producing megakaryocytes were rare, and platelets were only occasionally seen. No increase in blast cells was identified (0%). Bone marrow and peripheral blood morphology are shown in **Figure 1**. Conventional flow cytometry did not reveal any aberrant immunophenotype.

Conventional G-banding karyotyping was performed on unstimulated bone marrow cells after 24-hour culture (banding resolution 200–300). The result was 46,XY,t(5;14)(q31;q24) (20), with all 20 metaphases showing the same translocation and no additional clonal abnormalities detected (**Supplementary Figure 1**). FISH with *PDGFRB* break-apart probes was not performed.

**Figure 2:**
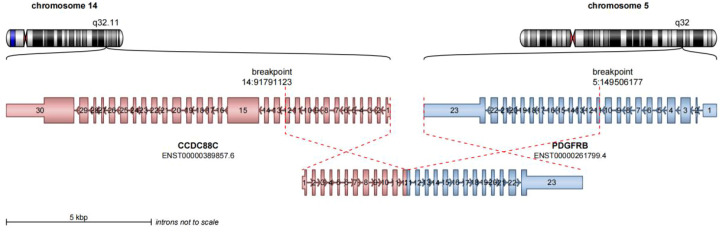
Schematic representation of the *CCDC88C::PDGFRB* fusion identified by RNA sequencing. The fusion involves exon 12 of *CCDC88C* and exon 11 of *PDGFRB*, resulting from a t(5;14)(q32;q32) translocation (at nucleotide resolution). The chimeric protein retains the N-terminal coiled-coil domain of *CCDC88C* and the C-terminal tyrosine kinase domain of *PDGFRB*, leading to constitutive kinase activation.

Whole transcriptome RNA sequencing was performed on bone marrow RNA using ribosomal RNA depletion (RiboZero) library preparation and the Illumina NovaSeq 6000 platform. Fusion genes were detected using the STAR-Fusion pipeline with the hg19 reference genome. RNA sequencing identified an in-frame *CCDC88C exon 12::PDGFRB* exon 11 fusion (hg19: chr14:91791123 to chr5:149506177), supported by 72 junction reads and 6 spanning reads (**Figure 2**). The fusion retained the C-terminal tyrosine kinase domain of *PDGFRB* (7, 8).

Whole exome sequencing (WES) and low-depth whole genome copy number sequencing (CNV-seq) were performed for additional molecular characterization. WES covered >20,000 protein-coding genes and flanking regions at a mean depth exceeding 2000×, detecting single nucleotide variants and small insertions/deletions. CNV-seq covered all autosomes and sex chromosomes, capable of detecting chromosomal aneuploidies and copy number alterations ≥100 kb. No additional chromosomal abnormalities were identified beyond the known t(5;14) translocation. WES detected a heterozygous ATM frameshift mutation, c.1381del (p.Glu461LysfsTer12), with a variant allele frequency of 35.7%, classified as likely pathogenic by ACMG criteria. Germline origin was not formally confirmed by testing non-hematopoietic tissue. No pathogenic or likely pathogenic variants were identified in CSF3R, consistent with a pure *PDGFRB*-rearranged neoplasm. Based on these findings, a diagnosis of *PDGFRB*-rearranged myeloid neoplasm was established. Imatinib was initiated at 200 mg once daily on January 23, 2026. This dose was selected based on evidence that low-dose imatinib (100–200 mg/day) is sufficient for sustained remission in *PDGFRB*-rearranged neoplasms (9, 11). Jawhar et al. (9) reported that 7 of 22 patients with *PDGFRB*-rearranged myeloid/lymphoid neoplasms achieved complete molecular remission on 100 mg/day, and Barbato et al. (7) similarly used 200 mg/day with complete molecular remission in a PDGFRB::CCDC88C-rearranged case.

**Figure 3:**
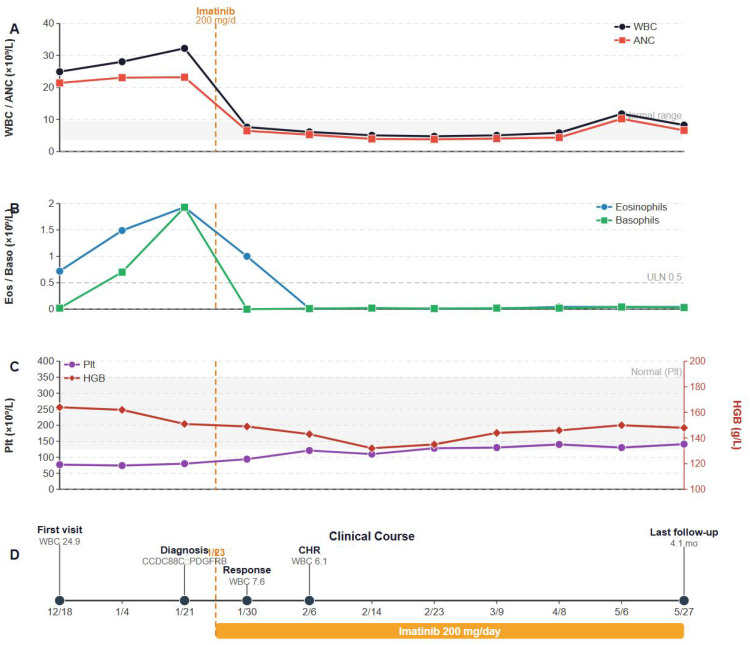
Hematologic parameters and clinical timeline. **(A)** WBC and ANC. **(B)** Eosinophil and basophil counts. **(C)** Platelet count and hemoglobin (dual-axis). **(D)** Clinical timeline. The orange dashed line indicates imatinib initiation (200 mg/day).

A rapid hematologic response was observed, with the WBC count decreasing to 7.61 × 10^9/L by January 30, 2026, and further normalizing to 6.10 × 10^9/L by February 6, 2026. Platelet counts gradually recovered, and eosinophil levels normalized. As of the last follow-up on May 27, 2026, blood counts remained normal, indicating durable hematologic remission lasting approximately 4 months. The dynamic changes in hematologic parameters are shown in **Figure 3**.

## Discussion

Myeloid/lymphoid neoplasms with *PDGFRB* rearrangements are characterized by constitutive activation of tyrosine kinase signaling and are typically associated with eosinophilia and sensitivity to imatinib (1, 3). Nevertheless, clinical heterogeneity is increasingly recognized, and atypical cases without prominent eosinophilia may pose diagnostic challenges.

In this case, a rare *CCDC88C::PDGFRB* fusion was identified. Only a limited number of such cases have been reported, highlighting the rarity of this fusion partner (6, 7). Similar to previously published cases, the fusion in our patient was associated with sensitivity to imatinib. Compared with earlier reports, however, our patient was notable for an asymptomatic presentation with predominant neutrophilia and only mild eosinophilia, further broadening the clinical spectrum of *PDGFRB*-rearranged neoplasms (5, 7).

The oncogenic mechanism of *PDGFRB* fusion proteins is primarily driven by constitutive activation of the kinase domain. In most cases, the 5′ fusion partner provides an oligomerization domain, enabling ligand-independent receptor dimerization and activation (1, 3, 7). *CCDC88C* encodes a coiled-coil domain-containing protein involved in cytoskeletal organization and Wnt signaling regulation. The presence of oligomerization-related domains in *CCDC88C* may facilitate constitutive activation of *PDGFRB* kinase signaling. Activated *PDGFRB* signaling can trigger multiple downstream pathways, including *STAT5*, *PI3K/AKT*, and *MAPK* pathways, thereby promoting proliferation and survival of myeloid cells (1, 3).

A notable feature of this case was not the complete absence of eosinophilia, but rather its mild nature. Although eosinophilia is a common feature of *PDGFRB*-rearranged neoplasms, it is not universal (1, 2). The predominance of neutrophilia in our patient suggests that additional molecular or lineage-specific factors may influence phenotypic heterogeneity. Such atypical presentations may lead to misdiagnosis as other myeloproliferative neoplasms, emphasizing the importance of comprehensive molecular testing.

The rapid hematologic response to low-dose imatinib strongly supports the pathogenic role of the fusion. *PDGFRB*-driven neoplasms are highly sensitive to tyrosine kinase inhibition, and early molecular identification of such fusions is therefore critical for guiding targeted therapy (1, 7).

A concurrent *ATM* frameshift mutation was also identified. Although *ATM* is a key regulator of the DNA damage response and genomic stability, its role in *PDGFRB*-rearranged neoplasms remains unclear. In this context, the *ATM* variant detected in this case warrants cautious interpretation. With a VAF of 35.7% and no confirmation from non-hematopoietic tissue, its origin remains uncertain—it could be somatic, germline, or mosaic. The patient had no personal or family history suggestive of ataxia-telangiectasia. Whether this variant contributes to the disease phenotype is unknown (10).

Importantly, this case highlights the diagnostic value of RNA sequencing in detecting rare gene fusions. Notably, the karyotypic localization of the 14q breakpoint to band q24 differs from the molecular localization of *CCDC88C* at 14q32.11. This discrepancy reflects the inherent resolution limit of conventional G-banding (approximately 5–10 Mb at the 200–300 band level), rather than a biological inconsistency. RNA-seq maps breakpoints at nucleotide resolution and is the gold standard for fusion partner identification in this context. Conventional cytogenetics and morphology may be insufficient to identify actionable kinase fusions, whereas comprehensive molecular profiling enables precise diagnosis and personalized treatment (4). Molecular testing is also essential to distinguish *PDGFRB*-rearranged neoplasms from other *BCR::ABL1*-negative myeloproliferative neoplasms, as well as from cytogenetically similar but biologically distinct fusions that are not sensitive to tyrosine kinase inhibitors.

Six cases of *CCDC88C::PDGFRB* fusion (including one in the inverted *PDGFRB::CCDC88C* orientation) have been reported across five publications (**Supplementary Table 1**) (6–8, 11, 12). The breakpoints are heterogeneous, involving at least five different exon–exon junctions, and the associated diagnoses range from chronic myeloid/lymphoid neoplasms with eosinophilia to *BCR::ABL1-like* acute lymphoblastic leukemia. Imatinib was effective in every case, consistent with retention of the *PDGFRB* tyrosine kinase domain across all reported fusions. The present case differs from previously reported cases in three respects: a previously undescribed fusion breakpoint (*CCDC88C* exon 12 → *PDGFRB* exon 11), the oldest patient to date (60 years), and the first concurrent germline *ATM* pathogenic variant.

## Limitations

Several limitations of this report should be acknowledged. First, FISH with *PDGFRB* break-apart probes and RT-PCR validation of the fusion transcript were not performed, though the combination of conventional karyotyping and RNA-seq provided sufficient evidence for diagnosis. Second, fusion transcript monitoring by RT-qPCR was not performed during follow-up; this would be valuable for assessing molecular response depth and guiding treatment duration, analogous to *BCR::ABL1* monitoring in CML. Third, the follow-up duration is relatively short (approximately 4 months), and extended observation is needed to confirm the durability of remission and identify any potential late relapse or resistance. Finally, whether the germline *ATM* pathogenic variant contributed to the disease phenotype or carries prognostic significance remains uncertain.

## Conclusions

This case expands the clinicopathologic spectrum of *CCDC88C::PDGFRB*-rearranged myeloid neoplasms by demonstrating that prominent eosinophilia is not required for diagnosis. Comprehensive molecular testing, particularly RNA sequencing, is essential in atypical myeloproliferative presentations, as timely identification of actionable kinase fusions can directly guide effective targeted therapy.

## Data Availability

The original contributions presented in the study are included in the article/**Supplementary Material**. Further inquiries can be directed to the corresponding author.
