# Supplementary material for: CCDC88C::PDGFRB-rearranged myeloid neoplasm with predominant neutrophilia and rapid response to imatinib: a molecularly defined case report
Source: Front Oncol. 2026 Jun 22;16:1855322. doi: 10.3389/fonc.2026.1855322 (PMC13333393; doi:10.3389/fonc.2026.1855322)
Supplement: Supplementary file 1 [file DataSheet1.pdf]

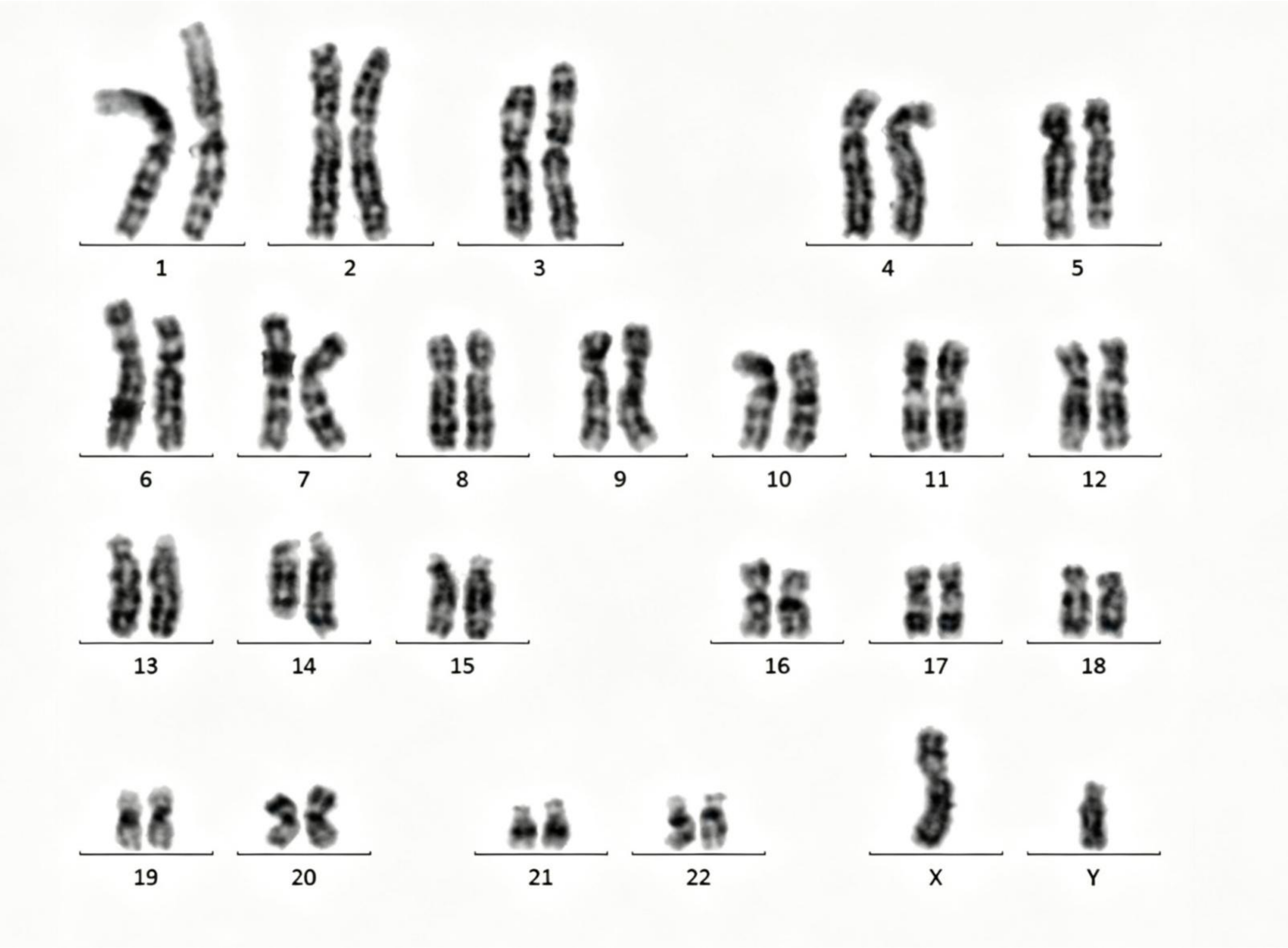


**Figure S1. G-banded karyotype: 46,XY,t(5;14)(q31;q24)[20].**

**Table S1: Clinical Summary of Reported *CCDC88C*::*PDGFRB* Fusion Cases**

| # | Study (Year) | Age/Sex | Diagnosis (WHO 2022) | Key Features | Fusion Breakpoint | Additional Findings | Treatment | Response | Follow-up | Ref |
|---|---|---|---|---|---|---|---|---|---|---|
| 1 | Gosenca et al. (2014) | NR | MLN-eo | NR | *CCDC88C* ex10 → *PDGFRB* ex12 | NR | Imatinib (NR) | CHR | Durable | [8] |
| 2 | Gosenca et al. (2014) | NR | MLN-eo | NR | *CCDC88C* ex25 → *PDGFRB* ex11 | NR | Imatinib (NR) | CHR | Durable | [8] |
| 3 | Bielorai et al. (2019) | 2.5 y/M | MPD + T-LBL | NR | *CCDC88C/PDGFRB* (NR) | t(5;14)(q33;q32) | Imatinib (NR) | Sustained response | RT-qPCR monitored | [11] |
| 4 | Oya et al. (2021) | 22 y/F | *BCR*::*ABL1*-like B-ALL | Relapsed post-chemo | *CCDC88C/PDGFRB* (NR) | NR | Inotuzumab → Imatinib + IT MTX | MRD $10^{-2}$→$10^{-3}$; fusion cleared | Planned HSCT | [12] |
| 5 | Tao et al. (2026) | Med 8 y (n=7) | *PDGFRB*-ALL | NR | *CCDC88C/PDGFRB* (NR) | *IKZF1*, *EBF1*, *PAX5*, *CDKN2A/B* (6/7) | Chemo ± TKI | CR 100% (7/7) | NR | [6] |
| 6 | Barbato et al. (2026) | 21 y/M | MLN-TK | Eos+basophilia, splenomegaly | *PDGFRB* ex1–12 → *CCDC88C* ex10–22 (inverted) | None (30-gene NGS) | Imatinib 200 mg/d | CMR (fusion cleared) | Ongoing | [7] |
| 7 | Present case | 60 y/M | MLN-TK | Neutrophilia, mild eos, thrombocytopenia | *CCDC88C* ex12 → *PDGFRB* ex11 | *ATM* germline fs; del(5)(q32) | Imatinib 200 mg/d | CHR | Ongoing (4 mo) | — |

**Abbreviations:** MLN-eo = myeloid/lymphoid neoplasm with eosinophilia; MLN-TK = myeloid/lymphoid neoplasm with tyrosine kinase gene fusion; MPD = myeloproliferative disorder; T-LBL = T-lymphoblastic lymphoma; B-ALL = B-cell acute lymphoblastic leukemia; ALL = acute lymphoblastic leukemia; CHR = complete hematologic remission; CMR = complete molecular remission; CR = complete remission; MRD = minimal residual disease; HSCT = hematopoietic stem cell transplantation; IT = intrathecal; MTX = methotrexate; NR = not reported; fs = frameshift; NGS = next-generation sequencing.
